# Analysis of Drag Reduction Methods and Mechanisms of Turbulent

**DOI:** 10.1155/2017/6858720

**Published:** 2017-09-18

**Authors:** Gu Yunqing, Liu Tao, Mu Jiegang, Shi Zhengzan, Zhou Peijian

**Affiliations:** ^1^College of Mechanical Engineering, Zhejiang University of Technology, Hangzhou 310014, China; ^2^School of Energy and Power Engineering, Jiangsu University, Jiangsu, Zhenjiang 212013, China

## Abstract

Turbulent flow is a difficult issue in fluid dynamics, the rules of which have not been totally revealed up to now. Fluid in turbulent state will result in a greater frictional force, which must consume great energy. Therefore, it is not only an important influence in saving energy and improving energy utilization rate but also an extensive application prospect in many fields, such as ship domain and aerospace. Firstly, bionic drag reduction technology is reviewed and is a hot research issue now, the drag reduction mechanism of body surface structure is analyzed, such as sharks, earthworms, and dolphins. Besides, we make a thorough study of drag reduction characteristics and mechanisms of microgrooved surface and compliant wall. Then, the relevant drag reduction technologies and mechanisms are discussed, focusing on the microbubbles, the vibrant flexible wall, the coating, the polymer drag reduction additives, superhydrophobic surface, jet surface, traveling wave surface drag reduction, and the composite drag reduction methods. Finally, applications and advancements of the drag reduction technology in turbulence are prospected.

## 1. Introduction

With the rapid increase of energy consumption, energy problem has been a global issue, which must be faced with and solved. Energy conservation and consumption reduction become an important research project at home and abroad. Drag reduction technology is urgently needed in transportation and military fields to reach the energy-saving goal. It is indicated by investigation and research that when the ship is sailing under water, the frictional force of its surface can account for 70% ~ 80% of total resistance. Even in the case of high-speed operation, its frictional force can also account for about 40% [1]. It can be calculated that in the case of certain power and energy, when the resistance decreases by 10%, the ship's speed and distance will increase by about 3.57% [2]. When the Reynolds of fluid in a round pipe reaches 10^5^ and the turbulence intensity is 10%, Reynolds turbulent stress will be about 100 times as much as laminar viscous stress and the greater the stress is, the greater the loss of energy is, there will be a lot of energy consuming in the flow friction [3]. In a sense, lot of energy consumption will be saved every year, even by slightly reducing the sailing resistance under water, which is of great significance for easing the energy crisis in the case of relative energy shortages nowadays.

Based on this, people always devote themselves to researching drag reduction methods. Natural creatures form a series of biological structure adapting to drag reduction after years of evolution. In recent years, scientists have found that the surface structures of many creatures are nonsmooth, the skins of sharks and dolphins have distributed many flake-like rib structures [4], which can change the skin's surface structure of turbulent layer and velocity distribution when the shark is swimming [5], while the earthworm's back can secrete some milk-white liquids, thus having wetting function and being able to reduce soil adhesion to the animal's body surface, which can research the goal of drag reduction [6]. These conditions of drag reduction on natural creatures give us great insights. Additionally, people also reach and develop many drag reduction methods, which provide many kinds of ways and means, attaining a series of achievements.

## 2. Bionic Drag Reduction Technology

### 2.1. Bionics

Bionics is an emerging discipline which can produce bionic productions, approximately with some characteristics of biological surfaces by imitating biological systems or using artificial technology to design and optimize productions for the biological function itself and to process for many aspects in function, shape, structure, material, and so on [7]. Natural creatures have developed into many kinds of biological bodies adapting to the environment in order to meet the need of living. These structures, shapes, and other relevant factors form the maximal ability to adapt to and coordinate the living environment by optimizing and coupling [8, 9]. Bionics was first born in the West in 1960. At an early stage, people understood and imitated natural creatures to apply this in practical production and life. As early as 2000 years ago in China, people began to imitate natural creatures consciously, for example, the famous carpenter in ancient times, Lu ban, invented saw for lumbering by observing the jagged edges of blades grass. In today's world, bionics has undergone rapid development, some research achievements have applied in people's daily life, for example, ultrasonic testing was invented by researching the bat [10], and riblet surface drag reduction was proposed by observing the shark when swimming [11].

### 2.2. Biological Surface Structures

#### 2.2.1. Shark Skin's Nonsmooth Surface

**Figure 1:**
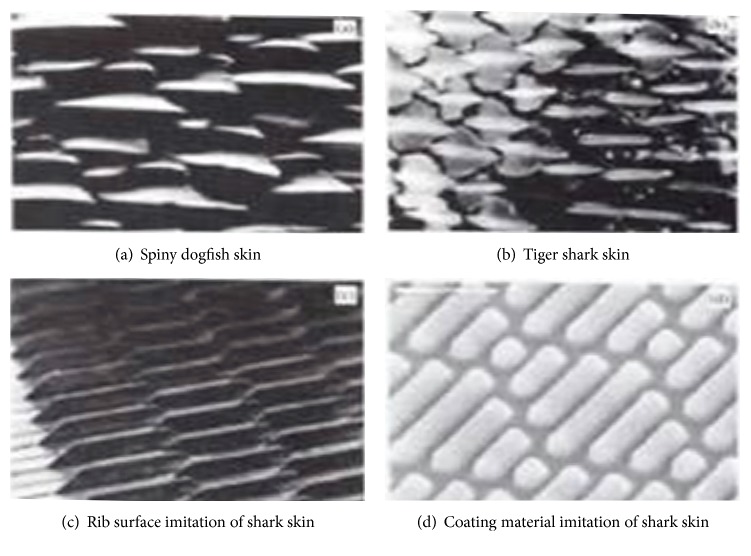
Shark skin microstructure and its imitation.

The shark is one of the fastest swimming animals in the ocean, whose skin surface does not adhere to any halobios, having an excellent drag reduction effect. By long-term observation for sharkskin, its surface is not smooth but is composed of many scales with grooved shape, with more spines and setae [12]. Sharkskin's microstructure and its mimic are shown in Figure 1 [13]. The scales of sharkskin are shield scales, whose configuration are compact, orderly, and jagged. The middle teeth are long, while the side teeth are short. The tooth spaces are toward the shark's tail direction and have overlapping phenomenon. Lot of researches reveal that the special structure of sharkskin mainly has two aspects of the role: on the one hand, this structure has antifouling function, decreasing adhesion on skin by other benthos; on the other hand, it is in favor of drag reduction in swimming. The cause of drag reduction is that this structure can change the intrinsic structure and the velocity distribution in turbulent boundary layer when the shark is swimming, which has a good effect on drag reduction.

#### 2.2.2. Earthworm's Nonsmooth Skin

**Figure 2:**
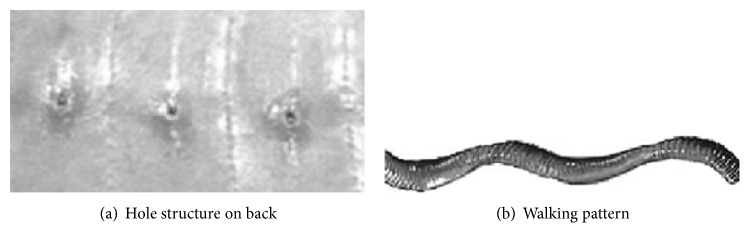
Earthworm's body surface.

The earthworm is a kind of soft animal, which has the characteristic of reducing adhesion to move freely without soil adhesion in clay soil. Through the biomechanical angle of earthworm crawling, the frictional resistance between the body and the soil surface was analyzed. The characteristic of an earthworm is the result of coupling with many factors, for example, earthworm's nonsmooth skin, its soft figure, and special way of movement [14, 15]. Earthworm's skin structure is shown in Figure 2() [6]. The back hole of the earthworm is concave pit on a nonsmooth surface structure. It can secrete some milk-white liquids and wet the surroundings, which can form a lubricated interface between soil and earthworm's body, reducing the adhesion of soil for the creature's skin.

#### 2.2.3. Dolphin's Flexible Body Surface

The mystery of high-speed and low resistance, when the dolphin is swimming, is always one of the hot research issues for all countries' scientists. The United States, Europe, and other countries have conducted plenty of theoretical studies and experimental researches, currently attempting to find the drag reduction mechanism of the dolphin's skin, which is mainly composed of two parts by research: for one thing, in the process of dolphin's swimming, its eyes can secrete a special liquid, which covers the whole skin coordinating with the streamline body shape carve of the dolphin. The liquid can reduce the friction force when the dolphin is swimming, having a lubricating function; another most key cause is that the dolphin can produce a wave in the process of swimming. It is produced from the dolphin's head, transferring to the tail at a very high frequency and minimum amplitude, changing the dolphin's skin into a flexible skin, which is very similar with the mechanism of flexible wall drag reduction. What is more, the dolphin's skin wave can produce vortex layer when the dolphin is swimming, which has an effect on drag reduction to some extent [16].

### 2.3. Bionic Drag Reduction Methods

#### 2.3.1. Groove Surface Drag Reduction

In the field of traditional thought, people always focus more energy on the smooth surface drag reduction. With the further research of bionics, people find that the microgrooved surface can effectively reduce the frictional force on the wall. At present, the practical applications of groove surface drag reduction mainly focus on 3 parts: aircraft, fluid drive apparatus, and pipeline fluid conveying equipment [17]. As early as the 1980s, German aircraft manufacturers began to use this technology. They found that through the technology, 8% of the fuel could save, when the plane flew the same distance. Currently, the United States, Europe, and other countries study deeply the groove surface drag reduction when the aircraft is flying and achieves great development.

By researching the groove surface drag reduction, there are mainly two aspects that influence its characteristic: its characteristic and the flow-field environment. The self-factor of groove surface includes its shape and size. The flow-field environment includes its pressure gradient, the shape of flow cross-section, and the fluid velocity. Researching a variety of geometries of groove (including V-shape, oval, semicircular, jug, and rectangular), the drag reduction effect of the V-shape groove is the best [18]. In the study of the placement of groove surface, the surface in distribution of the flow direction can effectively control the quantity of the low strip, while the distribution of lateral direction can inhibit the length of the low strip. There is a big difference between researchers about the influence of placement mode on resistance, and the result of experiment still has a big discrepancy.

**Figure 3:**
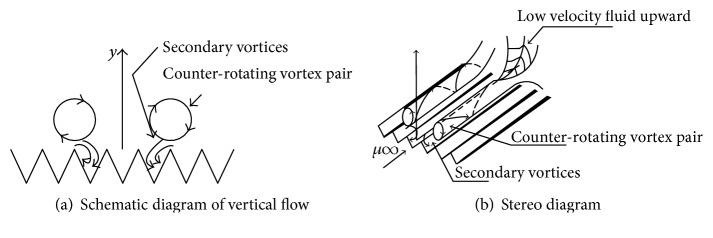
Schematic diagram of the interaction between the grooved surface and streamwise vortex.

The optimal drag reduction surface is not the smooth surface as is described by classic experiments [17]. Groove surface drag reduction is related to the turbulent flowing structure. We can make a summary as follows by researching its mechanism at present; considering wall shear stress simply from fluid mechanics perspective, the groove surface drag reduction mechanism owes to the increase of the vicious sublayer thickness. The surface has an obvious delay in the transition from laminar boundary layer to turbulent boundary layer [19], which can also change the turbulent characteristics in the near wall region [20–22]. From the theory of turbulent coherent structure, its mechanism is that the streamwise vortexes associated with low-speed strips are reduced and the spanwise gather of the low-speed strips is suppressed under the interaction between streamwise vortexes and secondary eddies, as shown in Figure 3, which is produced under the groove. The strip transition on the groove surface is more flat compared with the smooth surface. Low-speed strip transitions have a good linearity, revealing that the groove restrains the lateral fluid flow and strengthens the stability of fluid flow. From the principle of mechanical drag reduction, a similar “air bearing theory” can be put forward.

#### 2.3.2. Flexible Wall Drag Reduction

After a long-term study of dolphins and sharks, people find these marine organisms' skins are heavily elastic, which can reduce drug while they are swimming. According to this characteristic, scientists invent the flexible wall drag reduction method, daubing polymers on the solid wall and filling in their space with liquid to buffer external pressure with high elasticity. Both Cooper and Carpenter devote themselves to optimizing compliant coating to attain the best effect on drag reduction [22]. Russian scholars, Kulick and Semonov prove that the flexible wall has an important influence in reducing flow noise and surface frictional force by researching, the biggest drop up to 7%.

The flexible wall drag reduction mechanism is mostly increasing the thickness of the wall viscous sublayer, putting off the transition from laminar boundary layer to turbulent boundary layer and reducing the velocity gradient on the boundary layer, which results in reducing the shear force on the solid wall [23]. Even though, it still has a certain limitation, which is applicable to the high-speed and turbulent state. Its witnesses are exposed in low-speed flowing. Applying the flexible wall in the low-speed flow, it can reduce its own weight and deepen the depth of ship into the water. In this condition, its effect on drag reduction is not ideal.

## 3. Other Drag Reaction Methods

### 3.1. Microbubble Drag Reduction

**Figure 4:**
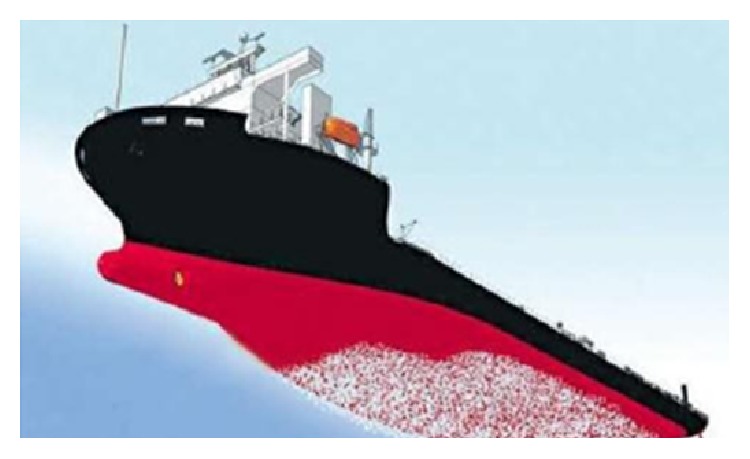
Bubble reduction ship.

Microdouble drag reduction is one of the main approaches in achieving jet drag reduction. With the depth of microair bubble drag reduction, it mainly applies in many fields, such as ship drag reduction, ultrasonic imaging, and sewage disposal. It is worth mentioning that microbubbles have an increasing effect on ship drag reduction, which becomes one of hot research issues. For a sailing ship, drag reduction can be achieved by covering the bubble layer on the hull. Reducing the density of the fluid medium with this method can change the boundary internal structure and the flowing kinematic and dynamic characteristics of the fluid in the near wall region, as shown in Figure 4. Meanwhile, we can take advantage of the small frictional force on the bubble surface and deformable regulating flow structure to reduce drag [24, 25]. Studies have shown that the drag reduction efficiency is up to 20% ~ 80%. Its mechanism is very complicated and affected by many factors, such as the number of hull surface, the size of bubbles, the structure of the hull, and flow parameter on the hull surface, which can affect the drag reduction effect [24]. For the hull surface, a gas-liquid mixture can be formed by injecting air, which can change the internal structure. Because of the volatility of the bubbles, partial work, produced by applying the shear force in the fluid, translates into deformation energy, reducing the loss of energy to the drag reduction effect.

**Figure 5:**
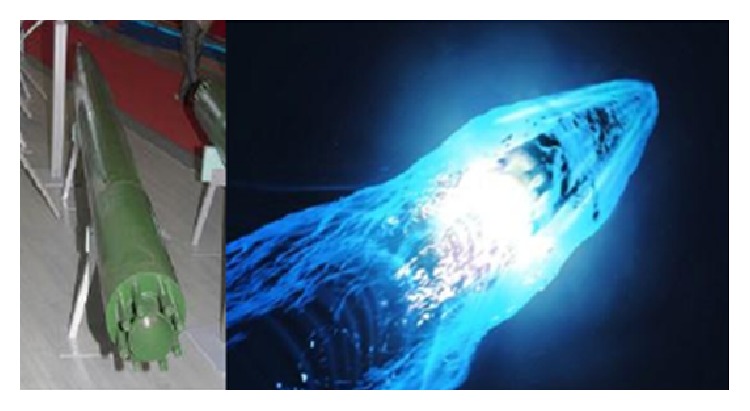
Supercavitating missile.

Presently, about the study of micro bubbles, the foreign scholar, Madavan et al., [26] researches the factors of influence in the microbubble drag reduction. Merkle researches the microbubble turbulent boundary layer effect of plat. The study relatively lags behind and starts late in our country. Based on the achievements of his predecessors, Wang [27] researches the theory of microbubble drag reduction mechanism using the Mac method axisymmetric body in computational fluid dynamics. Now, using the technology in the field of super gun shells can reduce the resistance, thereby increasing the speed of water missile as shown in Figure 5.

Similar with flexible wall drag reduction, though microbubble drag reduction has obtained a substantial progress, it also has many weaknesses. An obvious weakness is that the bubbles covering the hull surface are not steady and vulnerable to cracks to generate enlarged drag force and noise. If the bubbles are too small, the needed drag reduction cannot be achieved [24]. Besides, its another weakness is that when the ship is sailing, the microbubbles covering on its surface are mainly hydrogen and oxygen bubbles generated by electrolyzing water. Though the source of reactant is abundant and its resultants are not pollutional to the environment, the degree of demand for and dependence on electric energy is very high.

### 3.2. Vibrant Wall Drag Reduction

**Figure 6:**
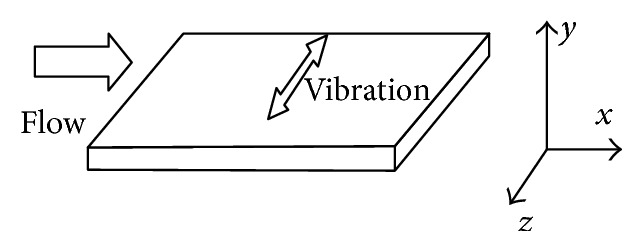
Schematic diagram of spanwise wall oscillation.

Relative to the previous several drag reduction method, wall vibration reduction is a relatively new drag reduction technology, which refers to the exhibition along the flow direction of the fluid flow through the smooth plate and vibration, which leads to a method of flat surface resistance decreased, as shown in Figure 6. Jung et al. verified the effectiveness of drag reduction about periodic spanwise vibration of smooth flat plate in 1992, using DNS means for the first time [28]. In recent years, foreign scholars have revealed the vibrant wall drag reduction mechanism by adopting DNS computational method and analyzing DNS data. There are two drag reduction mechanisms about the vibrant wall at present: first, the strips and vortexes generate an obvious incline, which can produce negative spanwise vortexes decreasing the mean velocity gradient in viscous sublayer and influencing the structure of the turbulent boundary layer. The streamwise vortexes rearrange along the spanwise, weakening the wave intensity of the streamwise vortexes in the lateral boundary layer, which is in favor of drag reduction. Another theory is that wall vibration interferes with the regenerative cycle of the quasi-streamwise vortexes, not sustaining wall turbulence, which can achieve the effect of drag reduction [22]. Because of the complexity of turbulence, there is no accurate expression about wall drag reduction at present and relevant researches need to be done in the future.

### 3.3. Coating Drag Reduction

With the rapid development of the economy, the demand for crude oil is increasing. Pipeline construction is imminent. In the long distance transportation of oil process, the friction drag between the oil and pipeline accounts for 98% of the total drag, which makes the power of the pump station almost entirely overcomes friction [29]. If the transportation drag of oil can be reduced, it can bring huge economic benefits. Based on this, the drag reduction problem in the pipeline has always puzzled researchers. With the development of coatings technology, coating in the pipeline as a mature technology has been applied in the long distance transportation of oil. The application of the coating in the pipeline can not only reduce the resistance, but also reduce the corrosion of the oil pipeline. As early as 1953, this method was applied in gas transportation in trunk pipeline of oil, which can increase the gas transportation capacity by 5% ~ 20% [30]. Wei and Ni discover the drag reduction effect of silicone oil is the best. The reason is the hydrophobicity of silicone oil can make the wall more smooth [29]. The hydrophobicity of coating and control for wall roughness is the essential reason of coating drag reduction.

The coating drag reduction mechanism mainly focuses on two aspects: smooth surface drag reduction and low surface energy drag reduction. In the pipeline process, because of the limitation of working accuracy, the pipeline always presents a certain degree of roughness and its surface is rugged. The flowing of oil in the pipeline is in turbulent state, which can form vortex area on the surfaces of concave-convex objects which can generate a certain pressure, bringing loss of energy which is related to the mean height of concave-convex objects. The higher the mean height is, the greater the loss of energy is. The coating on the tube wall can effectively smooth the wall to reduce the concave-convex height, which can reduce the mechanical drag produced by wall roughness. It is discovered by foreign relevant researches that the transition from laminar boundary layer to turbulent boundary layer can be put off by spraying compliant coating. For the low surface energy drag reduction, spraying compliant coating changes the wetting degree of the fluid on the tube wall because of the hydrophobicity of coating, reduces the velocity gradient of the fluid on the tube wall and, decreases the shear force on the wall, which can reach the goal of reducing the transportation drag. What is more, the difficulty that coating can be wetted is related to its drag reduction performance. The more difficult the coating can be wetted, the worse its drag reduction effect is.

### 3.4. Polymer Additive Drag Reduction

Besides the coating drag reduction, the drag reduction purpose also can be achieved by adding the polymer in the fluid. In the late 19th century, people found that muddy water can flow faster than clean water in some river sections. Later, they found that the frictional drag on the ship's surface is smaller when its sails in the water with water plants than without water plants [31]. In the late 1940s, Toms found the earliest drag reduction agent (DRA), polymethyl methacrylate (PMMA). Afterwards, researchers found other additives one after another. The DRA can be broadly divided into two categories: water-soluble and oil-soluble. Water-soluble DRA includes sesbania power, PAM (polyacrylamide), and synthetic PEO (Polyoxyethylene). Oil-soluble DRA includes olefin copolymer and polyisobutene [32].

In our country, though the study of DRA starts relatively late, great progress has still been made. In the 1980s and early 90s, Chengdu University of Science and Technology and Zhejiang University made a remarkable advance for the synthesis of indoor DRAs. The China Petroleum Pipeline Company made a breakthrough progress for the development of EP series oils, DRA, forming the independent intellectual property rights and starting mass production.

The polymer additives drag reduction is not still mature, but certain research results have been made. In 1967, Virk et al. discovered that there exists an elastic buffer area between laminar flow and turbulent flow in the near wall area, by measuring the fluid velocity before and after adding DRA, according to which, asymptote was being obtained. Early researchers consider that the existence of the elastic buffer area results in the enlargement of velocity and increase of flow in the pipe, attaining the drag reduction effect. Besides, Berman had a new discovery that the effect is better by injecting DRA than DRA is dissolved in liquid beforehand in 1986. Focusing on the influence of fluid kinetic characteristic, the polymer can change the turbulent structure on the wall. Abernathy drew two conclusions one is that polymer can impede the generation of eddies and reduce the frequency of vortex; the other is that polymer can reduce the rotation rate of the vortex. Both of them together can result in the drag reduction effect. Some hypotheses and analyses can be proposed combining with the experimental results for the polymer drag reduction mechanism, but there is still no unified theory which can explain all experimental phenomena [33, 34].

At the same time, artificial polymer can cause biological damage to the ship's hull, and some organic DRA can pollute the environment. So, the polymer drag reduction agent is commonly used for oil pipeline transportation, but it will add additional funding.

### 3.5. Superhydrophobic Surface Drag Reduction

**Figure 7:**
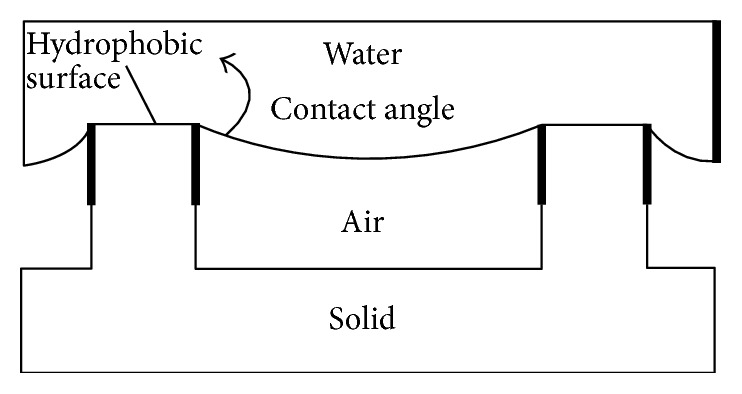
Schematic diagram of drag reduction mechanism of the superhydrophobic surface.

According to the phenomenon, water rolls easily without wetting on the lotus leaf because of the microscale structure of the emulsion on the lotus leaf rejecting the water, it invented the method of the superhydrophobic surface drag reduction [35]. So far, the mechanism accepted of the superhydrophobic surface drag reduction is the theory of slip length considering that because water produces the wall slip through hydrophobic surface decreasing velocity gradient of the boundary layer, decreasing the shear stress, and delaying the change of laminar attachment surface, the laminar flow regime tends to be more stable [36]. The mechanism of the superhydrophobic surface drag reduction is shown in Figure 7().

**Figure 8:**
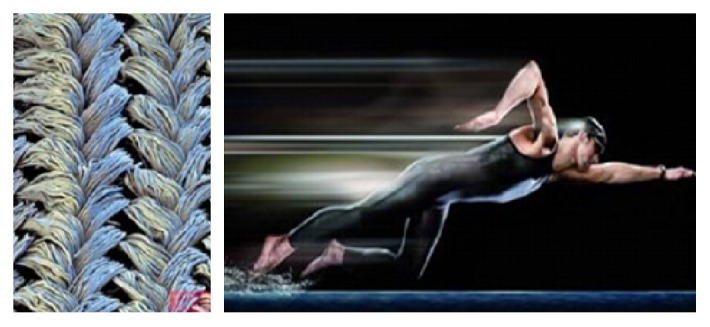
Bionic swimsuit.

Voronov et al. [37] found that by numerical simulation, while the slip length and contact angle are usually regarded as hydrophobic performance standards, they do not always follow the same rules, low *σ*_r_ (relative atomic size) tends to make the contact while the angle increases but will reduce the slip length. Bixler and Bhushan [38] obtained a maximum drag reduction rate of 26% by simulating shark skin resistance reduction experiments. Lee and Kim [39] had studied the applicable range of superhydrophobic drag reduction on hydraulic pressure. The present study found that the texture of the superhydrophobic surface captures the air to form the air cushion and reduces the frictional resistance between the fluid and the coating surface. Using the drag reduction characteristics of the superhydrophobic surface, the technique is applied to the surface of the swimming suit. As shown in Figure 8, this swimsuit is more conducive to increasing the speed of far mobilization compared to the imitation shark skin swimsuit.

### 3.6. Jet Surface Drag Reduction

The aim of jet technology is to control and provide power for itself through the interaction force of jet reaction and the interaction between jet and flow. It has short response time and good low voltage control characteristics [40].

**Figure 9:**
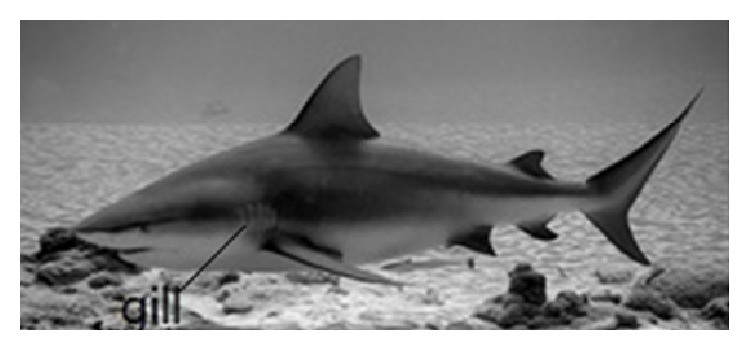
Feature of shark gills.

Sharks living in the sea are aquatic animals having rapid movement. The burst start speed of the deep-sea sharks is amazing, reaching up to 10 ~ 20 m/s; while pursuing their prey, they have very fast speed [41]. There are large gill plates lined in front the body side of sharks, as shown in Figure 9(), and each lamella has about 5 ~ 7 branchial cleft. As they swim, the water is sucked through the half opening mouth, and gas is exchanged from the gill slits. It is closely related with breathing and self-motion resistance reduction.

The researchers found the drag reduction effect on the surface of the jet according to the jet motion of the shark's gills. The present study generally believes that if there is jet on the motion face, jet fluid will block the mainstream field, forming the countercurrent area at the back of the jet hole and the direction of speed near the wall is contrary to flow velocity in the countercurrent area having the obvious drag reduction effect. Furthermore, there is the counter-rotating vortex extending downstream produced in the downstream of the jet hole and it would induce two vortex on the wall increasing the thickness of the boundary layer and decreasing the velocity gradient both having the effect of drag reduction [42].

To sum up, with the rapid development of science and technology, jet drag reduction technology has reached rapid development and fruitful achievements, many research results have been applied in the engineering practice, but there are still many problems that still need further research.

### 3.7. Traveling Wave Surface Drag Reduction

**Figure 10:**
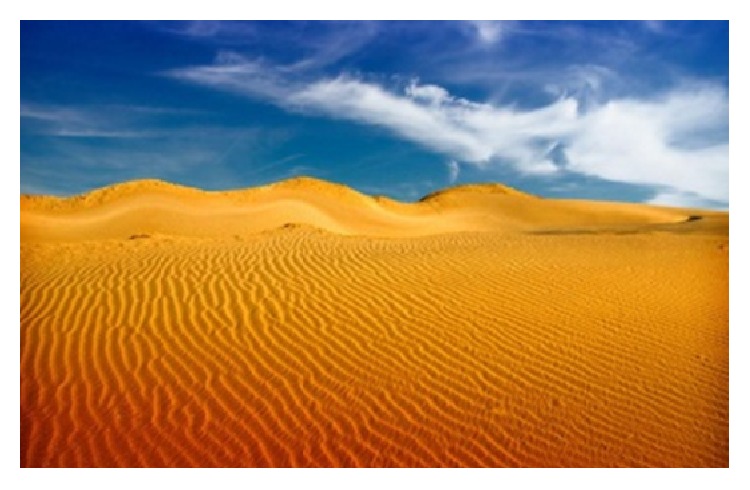
Wavy structures in the desert.

**Figure 11:**
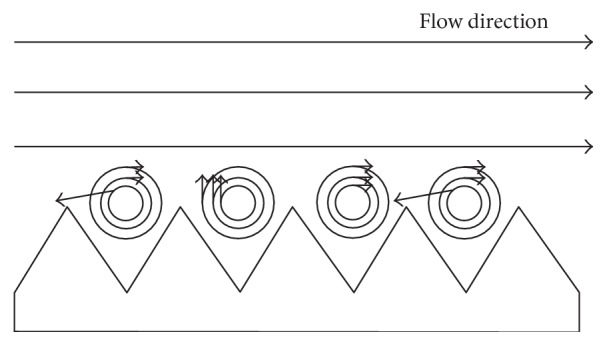
Schematic diagram of traveling wave.

Traveling wave surface drag reduction technology is considered a very promising drag reduction technology and is made of corrugated shape, which was inspired from the undulating dunes structure of desert, as shown in Figure 10. At present, the mechanism of drag reduction for traveling wave surface is still controversial. A more persuasive argument is that the ripples on the surface of the traveling wave shape can produce secondary flow, namely the origin parallel artificial vortex flow sparked a row, so that it is free to flow in the parallel artificial vortex flow, achieving the goal of drag and noise reduction. Its principle is shown in Figure 11.

### 3.8. Combined Drag Reduction

In practical engineering application, multiple drag reduction technologies would be used together achieving the best drag reduction effect. The effect of drag reduction using multiple drag reduction technologies is generally better than that using single drag reduction technique. It is the most universal using trench surface drag reduction and other drag reduction methods together in a variety of combinations. A series of turbulent drag reduction tests on coupling of trench surface and polymer coating technology have been carried out turning out that the effect of coupled drag reduction is excellent for the trench surface drag reduction and polymer coating drag reduction. Sun and found that this combination increases the stability of the wall air layer can make bubble not easy to break reducing the resistance and noise, so that the effect of drag reduction is optimized [11].

## 4. Summary and Outlook

The turbulent drag reduction technique a case turbulence theory applied to engineering has good development prospects. Due to the need of practical engineering, Chinese and foreigners have carried out a large number of experimental studies and gotten many achievements as references for practical application. However, most studies still stay in the simulation and test phase having not forming the mature systemic theory. In future research, the exploration of drag reduction mechanism should be put into more important place, especially by combining the numerical simulation and experimental study and focus on theoretical research guiding practice with theory. The drag reduction effect is greatly restricted, due to that, the research work of the current technology of drag reduction is mainly focused on single drag reduction technology. Therefore, to use multiple drag reduction technologies together will be an important direction for the future research. The research of technology is service for practice, so we should speed up the progress of promoting laboratory research results to the engineering application. And how to put the relatively mature drag reduction technology into actual projects and to promote it is also an important direction in the future research.
